# The implementation of HTA in medicine pricing and reimbursement policies in Indonesia: Insights from multiple stakeholders

**DOI:** 10.1371/journal.pone.0225626

**Published:** 2019-11-27

**Authors:** Riswandy Wasir, Sylvi Irawati, Amr Makady, Maarten Postma, Wim Goettsch, Talitha Feenstra, Erik Buskens

**Affiliations:** 1 Department of Epidemiology, University Medical Center Groningen, University of Groningen, Groningen, the Netherlands; 2 Sekolah Tinggi Ilmu Farmasi Makassar, Makassar, Indonesia; 3 Groningen Research Institute of Pharmacy, Faculty of Science and Engineering, University of Groningen, Groningen, the Netherlands; 4 Center for Medicines Information and Pharmaceutical Care, Faculty of Pharmacy, Universitas Surabaya, Surabaya, Indonesia; 5 Department of Clinical and Community Pharmacy, Faculty of Pharmacy, Universitas Surabaya, Surabaya, Indonesia; 6 National Health Care Institute, Diemen, the Netherlands; 7 Department of Pharmacoepidemiology and Clinical Pharmacology, Utrecht University, Utrecht, the Netherlands; 8 Department of Health Sciences, University of Groningen, Groningen, The Netherlands; 9 Department of Economics, Econometrics & Finance, Faculty of Economics & Business, University of Groningen, Groningen, the Netherlands; 10 Department of Pharmacology and Therapy, Faculty of Medicine, Universitas Airlangga, Surabaya, Indonesia; 11 Dutch National Institute for Public Health and the Environment (RIVM), Bilthoven, the Netherlands; 12 Department of Operations, Faculty of Economics & Business, University of Groningen, Groningen, the Netherlands; Medical University Graz, AUSTRIA

## Abstract

**Objectives:**

This study aimed to identify the barriers and facilitators to improve the use of health technology assessment (HTA) for the selection of medicines listed in the e-Catalogue and the national formulary in Indonesia.

**Methods:**

Semi-structured interviews were conducted to collect qualitative data. Purposive sampling was used to recruit the stakeholders consisting of policymakers, a pharmaceutical industry representative, healthcare providers, and patients. The data were analyzed using directed content analysis and following the COnsolidated criteria for REporting Qualitative studies (COREQ).

**Results:**

The twenty-five participants interviewed agreed with the use of HTA for supporting the e-Catalogue and the national formulary and perceived the advantages of HTA implementation outweighed the disadvantages. Barriers mentioned were a lack of capability of local human resources, financial incentives, a clear framework and insufficient data. Strategies suggested to overcome the barriers were establishing (inter)national networks to build up capacity, setting up departments of HTA in several universities in Indonesia, and introducing a clear HTA framework. Facilitators mentioned were the ambition to achieve universal health coverage, the presence of legal frameworks to implement HTA in the e-Catalogue and the national formulary, and the demands for appropriate medicine policies.

**Conclusions:**

Several barriers are currently hampering broad implementation of HTA in medicine pricing and reimbursement policy in Indonesia. Solutions to these issues appear feasible and important facilitators exist.

## Introduction

The 2015 transition from Millenium Development Goals to Sustainable Development Goals has triggered a shift in global health from service-specific targets to broader health system goals [1]. Target 3.8 of the Sustainable Development Goals explicitly states to achieve Universal Health Coverage (UHC) [2]. UHC will be accomplished when all people receive the healthcare services they need of sufficient quality and without suffering financial hardship. Therefore, the presence of UHC ideally will reduce or eliminate the proportion of out-of-pocket payments from healthcare expenditures [3]. Out-of-pocket payment, a direct payment to the healthcare providers at the time of service use, can drive an individual or a household below the poverty line [4]. In low middle-income countries (LMICs), the proportion of out-of-pocket payments in healthcare expenditures is still high, particularly for medicines, the number ranges from 50% to 90% [5].

Appropriate medicine policies combined with an implementation of health technology assessment (HTA) can facilitate countries to reduce the out-of-pocket payments for medicines on their way towards UHC. For instance, the United Kingdom (UK) and Thailand, as one of the oldest and the newest UHC examples respectively, have shown that the implementation of HTA supports their medicine policies [6]. Furthermore, the World Health Organization (WHO) highly recommends the use of HTA to faciltate the creation of a list of medicines in the medicine benefit package [7]. The WHO defines HTA as the systematic evaluation of properties, effects and/or impacts of health technologies and interventions [8]. HTA is a critical component of evidence-based policy decision making [7,9].

In response to target 3.8 of the Sustainable Development Goals, the Government of Indonesia launched a new national health insurance system, which is called *Jaminan Kesehatan Nasional–Kartu Indonesia Sehat* (JKN-KIS) in 2014. The JKN-KIS aims to achieve UHC by 2020 and is managed by Indonesia’s National Healthcare Security Agency, namely *Badan Penyelenggara Jaminan Sosial Kesehatan* (BPJS-Kesehatan) [10,11]. Additionally, several new medicine policies were introduced separately by the Ministry of Health to support the JKN-KIS. First, the e-Catalogue was introduced as a national medicine pricing policy. The e-Catalgue provides a list of medicines with specifications, prices, and suppliers. Second, the national formulary was compiled as a list of medicines covered by the BPJS-Kesehatan [12]. The e-Catalogue and the national formulary have their own respective responsible committees, which were both established in 2013, a year before the implementation of the JKN-KIS. Although the e-Catalogue and the national formulary were established separately, in practice these policies are interrelated. Indonesian healthcare facilities can reimburse medicines listed in the national formulary based on its prices listed in the e-Catalogue [12–14].

According to the national health insurance guidelines of Indonesia, an HTA approach should be used to select a health technology or intervention, including medicines, which will be covered by the BPJS-Kesehatan. This implies that HTA should be used in further developing the e-Catalogue and the national formulary. As a way forward, both may build on the guideline on the implementation of HTA in particular for medicines that the Department of Pharmacy and Medical Devices in the Ministry of Health of Indonesia introduced in 2013 [15]. In detail, the national health insurance recommended that the use of HTA is developed and performed by an HTA committee installed by the Ministry of Health [12].

Indeed in 2014, several months after the implementation of the JKN-KIS, the Ministry of Health formed the HTA committee. The committee had eight senior health scientists and six employees of the Ministry of Health, and was supported by 14 secretaries. The tasks of the committee were establishing a policy concept, guidelines, and the HTA committee itself to regulate the implementation of HTA [16]. In 2016, the HTA committee was reformed. The current HTA committee is composed of eight senior health scientists, one employee of the Ministry of Health, and supported by four secretaries. A technical staff with thirteen clinicians, two Ministry of Health employees, and one technician is now added to support the HTA committee tasks. Their current tasks are to define a guideline for HTA implementation, to establish the HTA committee and their work plan, to build a relationship with HTA committees in other countries, to assess the technologies or interventions covered by BPJS-Kesehatan, and to disseminate the results of their assessments [17].

However, although the HTA guideline was published in 2013 and the HTA committee had been installed in 2014, HTA has not been used in the development of the e-Catalogue and the national formulary. This might be one of the reasons why the use of the e-Catalogue and the national formulary could not help to reduce out-of-pocket payments for medicines in Indonesia [18]. In 2014, the out-of-pocket payments comprised approximately half of the total health expenditure in Indonesia, and it has remained at a similar level since [19]. The proportion of out-of-pocket payments for medicines was 70% in recent years [20]. Furthermore, over the same period, the Ministry of Health reported that up to 40% of prescribed medicines were not listed in the NF [21]. Relevant stakeholders were interviewed to provide a better understanding of how to improve the implementation of HTA in Indonesia, with particular attention for suggestions on and barriers perceived regarding the use of HTA in revising the e-Catalogue and the national formulary.

## Materials and methods

Semi-structured interviews with multiple stakeholders were conducted to collect qualitative data. The COnsolidated criteria for REporting Qualitative studies (COREQ) checklist was followed in reporting the results [22].

### Study design and participants

**Fig 1:**
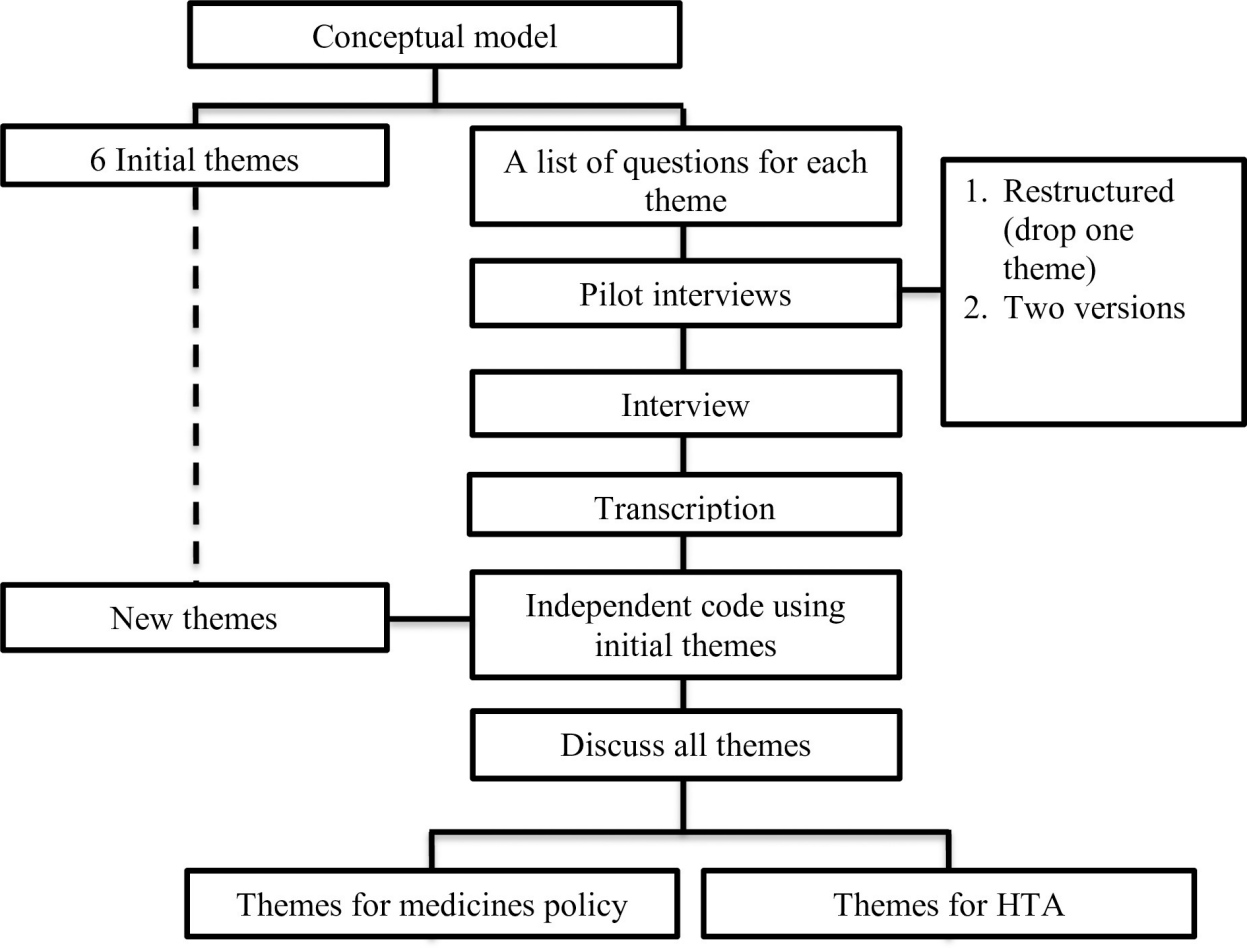
Research process.

The process applied in this study can be seen in the flowchart (Fig 1). First, a conceptual model (S1 Fig) was developed by RW, MP, WG, TF, and EB, based on a review of WHO documents on UHC, in particular concerning medicine policies and the use of HTA. Based on the conceptual model, initial list of themes and of questions (S1 Table) were created to get an overview of the development of medicine policies and the implementation of HTA in Indonesia. With the aim of testing the comprehensibility and appropriateness of the interview protocol (S1 File), pilot interviews were conducted with three physicians, three pharmacists, and three patients. After revision based on the pilot, semi-structured interviews were then conducted with various stakeholders. Interviews were planned until saturation was reached.

A purposive sampling method was used to recruit stakeholders, which consisted of policymakers, medicine suppliers, healthcare providers, and patients in various places of Indonesia. Recruitment criteria were: stakeholders (policy makers, healthcare provider, industry representative) should have at least 5 years of relevant work experience. Physicians were not selected based on their specialization, because HTA would be implemented in the development of the national formulary, which is used by all healthcare providers regardless of their specialization. Both pharmaceutical industries approached produced medicines and medical devices and were responsible for distributing medicine to the healthcare facilities. Patients were selected based on the duration of their health insurance in Indonesia (at least 5 years) and their routine use of medicines (at least 5 years). In order to ensure that these patient criteria were met, participants were recruited from the BPJS-Kesehatan’s chronic disease management program (*Program pelayanan penyakit kronis*, Prolanis).

A priory estimation of the number of participants required to reach saturation was made. We estimated that at least twenty-two stakeholders should be approached based on at least two representatives for each group of policy makers, i.e., at least two pharmaceutical industry representatives, at least four interviewees from each group of healthcare providers, and at least four patients. The reason for having more interviewees in the groups of healthcare providers and patients was our interest in the practical issues of the implementation of medicine policy and the difficulties reported for applying HTA for selecting medicine in the medicine policy. Potential participants were approached by email, phone and visit.

### Data analysis

All interviews were audiotaped and then transcribed. The transcripts were independently coded by two authors (RW, SI) using MAXQDA version 12.3.2. Directed content analysis [23] was applied to systematically structure the content of the transcripts. The codes were compared, and in case of disagreements, items were discussed with AM, WG, MP, TF, and EB to reach a consensus. Twenty four themes were identified during coding process. The themes were then divided into two categories. The first concerned medicine pricing and reimbursement policy [18] and the second the implementation of HTA. The current manuscript focused on the six themes (Table 1) related to the implementation of HTA.

**Table 1:** List of themes.

No	Themes
**1**	Attitudes towards HTA implementation
**2**	Advantages of HTA implementation in the e-Catalogue and the NF
**3**	Disadvantages of HTA implementation in the e-Catalogue and the NF
**4**	Barriers to HTA implementation
**5**	Possible solutions to improve the implementation of HTA
**6**	Promoting factors of HTA implementation

The transcript interviews from six themes were coded to attain sub-themes. Saturation at the total level was assessed to identify the sub-themes. Saturation was considered to be reached when no new information was generated and when at least three interviewees mentioned the same concern for each sub-theme. These were checked by RW and SI.

### Ethical considerations

The main author is Indonesian; therefore, an official research permit is not requested as stated in the relevant regulation of Indonesia”. However, a written informed consent (S2 File) was obtained from all participants. Before the interviews, all participants understood that their participation was voluntary and that they were free to stop the interview at any time. All participants reviewed and consented to the verbatim transcript of their interview. Additionally, the research plan and the interviews to be conducted were reviewed by the University Medical Center Groningen Ethical Review Board, who deemed the study non-intrusive. Subsequently a formal waiver statement (S3 File) was provided, i.e., the study would not need a regular ethical approval.

## Results

### Participants

Aiming at about 22 final participants, a total of 51 (S2 Table) different individuals were approached during the recruitment process. Out of this number, 45 agreed to participate in this research. However, 9 withdrew after receiving the list of questions; another 7 decided to stop their interviews before they were finished, since they experienced difficulties in answering the questions, and were not confident about their answer; Furthermore 4 had no time for an interview, leaving a final number of 25 participants. Notably, this was more than initially aimed at.

Twenty-five participants participated in this research (Table 2). They were policy makers (WHO members, HTA Committee, NF Committee and National Health Insurance Agency), a medicine supplier (a pharmaceutical industry representative), healthcare providers (physicians and pharmacists), and users (patients). All participants had the Indonesian nationality, except the WHO members. The policymakers, the pharmaceutical industry representative and healthcare providers each have more than 20 years of work experience in the Indonesian healthcare system. Furthermore, all interviewed patients had been enrolled in health insurance in Indonesia for an average of 11 years at the time of interview.

**Table 2:** Classification of participants involved.

Participants	Gender	Age (Years)	Work experience (Yes)	Years using public health insurance	Professional location
**Policymakers**					
WHO Members	Male	50	25	NA	Geneva
HTA Committee	Female	53	28	28	Jakarta
HTA Committee	Male	55	30	30	Jakarta
NF Committee	Male	56	31	31	Yogyakarta
JKN-KIS Agency	Female	51	26	26	Jakarta
JKN-KIS Agency	Female	46	21	21	Jakarta
**Medicine Supplier**					
Pharmaceutical Industry	Male	54	29	29	Jakarta
**Healthcare Providers**					
Physician	Male	58	33	33	Lombok
Physician	Male	59	34	34	Makassar
Physician	Male	66	41	41	Makassar
Physician	Male	44	19	19	Surabaya
Physician	Male	51	26	26	Jakarta
Physician	Female	54	29	29	Yogyakarta
Pharmacist	Female	53	28	28	Jakarta
Pharmacist	Male	50	25	25	Manado
Pharmacist	Male	39	14	14	Kendari
Pharmacist	Male	42	17	17	Yogyakarta
Pharmacist	Female	51	26	26	Lombok
Pharmacist	Female	40	19	19	Makassar
**Users**					
Patient	Male	53	NA	8	Makassar
Patient	Male	63	NA	13	Makassar
Patient	Female	59	NA	10	Makassar
Patient	Male	57	NA	8	Makassar
Patient	Female	63	NA	12	Makassar
Patient	Male	63	NA	13	Makassar

### Interviews and analysis of transcipts

Semi-structured interviews were conducted face to face with 16 participants and through video calls with 9 participants between August 2016 and April 2017. One interview was conducted in English, while all others were in Bahasa Indonesia. The average time spent on each interview was circa one hour.

Transcripts from the themes for HTA were checked to obtain sub-themes (S3 Table). For all six themes, more than three interviewees mentioned the same concern on each sub-theme. It means that saturation could be confirmed at the total level. In S3 Table we summarize the answers by respondent group, to make it easier for readers to understand the main concerns of each stakeholder group. The complete statement per theme can be found at raw materials (S4 Table).

### Findings per theme

#### Theme 1: Attitudes towards HTA implementation

All stakeholders demonstrated positive attitudes towards applying HTA to the development of the e-Catalogue and the NF. The main reason expressed for this attitude was the necessity of having appropriate medicine policies to support the JKN-KIS program in achieving UHC, in particular, reducing out-of-pocket payments for medicines. Moreover, participants perceived the advantages would outweigh the disadvantages.

“*If the HTA is applied to the NF or e-Catalogue I am very amenable*. *This will definitely be very good and provide great benefits”* [Physician 5]

#### Theme 2: Advantages of HTA implementation in the e-Catalogue and the NF

Stakeholders recognized that the main advantage of HTA is to provide scientific evidence for decision makers to assess the value of a medicine. Additionally, the participants identified various other benefits when HTA would be implemented in the e-Catalogue and the NF.

“*The money allocated for the health sector is limited*, *especially for medicines*. *So*, *we can convince the government that more money should be allocated for medicines”* [Policymaker 6]

The pharmaceutical industry representative mentioned that HTA would provide a ground for fair pricing in the e-Catalogue. Thus, pharmaceutical industries would not arbitrarily adapt their prices.

“*HTA can provide the rational price for bidding the medicines*. *Now*, *the pharmaceutical industry can bid the medicines as low as possibl”* [Pharmaceutical Industry]

The policy makers, healthcare providers, and patients mentioned the use of HTA would reassure all stakeholders that the medicines listed in the NF were the best choice of medication. This would convince the government to allocate the money for providing the medicines listed in the NF, the physicians to prescribe the medicines listed in the NF, and also the patients to consume the medicines listed in the NF.

“*The advantage is that we may not doubt anymore about taking the medicine listed in the NF”* [Patient 1]

#### Theme 3: Disadvantages of HTA implementation in the e-Catalogue and the NF

Among all interviewees, only a few could mention disadvantages of HTA implementation in the e-Catalogue and the NF. The most frequently mentioned disadvantage was the cost of developing the e-Catalogue and the NF probably would increase, in particular to pay HTA experts. In addition, complicated bureaucracy and lengthy processes for renewing the medicines listed in the e-Catalogue and the NF were also mentioned by the policy makers and healthcare providers.

“*People could say that this is too heavy or too bureaucratic*, *you need an excessively lengthy process for this”* [Policymaker 1]

#### Theme 4: Barriers to HTA implementation

The participants identified various barriers that are currently hampering the implementation of HTA. The first barrier mentioned by all stakeholder categories, except the patients, was a lack of capability and a lack of capacity of local human resources. The reasons for this as explained by several interviewees were that HTA is a new science in Indonesia, and a lack of HTA departments, training and associations.

*The science of HTA is still very new in Indonesia*. *Honestly*, *there are still many health workers and maybe including me who do not understand the application*.*”* [Pharmacist 5]

A second barrier mentioned by all categories of stakeholders, except the pharmaceutical industry representative, was a lack of incentives. The stakeholders considered that currently little resources are available for paying the HTA experts, holding HTA seminars, and performing HTA research.

“*There is a significant financial problem for the experts*, *since the HTA Committee still depends on the state budget and the standard fees established by the Ministry of Finance must be adhered to*. *We cannot give the fee according to their (HTA experts) expertise because there is a maximum salary that can be awarded when using the state budget”*. [Policymaker 3]

A third barrier mentioned by the policy makers pharmaceutical industry representative and healthcare providers was a lack of a clear framework of how to implement using HTA results in the medicine policy, in particular in the e-Catalogue and the NF. These interviewees mentioned that a clear framework is needed since multiple professions have to cooperate to initiate HTA, perform HTA, assess and appraise HTA results, and translate findings into policy advise.

“*We do not yet have a clear path of how to apply HTA*. *Moreover*, *HTA requires a variety of professions*. *This will create a conflict of interest*. *If there are no clear guidelines*, *all will be based on the point of view of each profession”*. [Pharmaceutical Industry]

A fourth barrier mentioned by the policy makers and healthcare providers was insufficient data for conducting HTA studies. The HTA committee members interviewed perceived the insufficient data was caused by difficulties to access data on the national scale. National data gathering is managed by the JKN-KIS agency. The JKN-KIS agency representative explained that the insufficient data was caused by unclarities regarding the data needed for conducting HTA studies. Furthermore, the health care providers also mentioned that the healthcare registry data, for instance, individual patient data, have not been integrated in the national scale.

“*The number of provinces in Indonesia makes it difficult to integrate all the data that could be used for HTA studies*, *so*, *we still need time to collect the data needed by the HTA researchers”* [Policymaker 6]

#### Theme 5: Possible solutions to improve the implementation of HTA

The participants provided a variety of possible solutions to address the barriers hampering the implementation of HTA. The policy makers indicated a necessity to establish a good network to build up the capacity. For instance, students and researchers could be endorsed to conduct HTA studies using Indonesian data. Currently, the Ministry of Health only depends on the state budget to establish the HTA committee and to build the capacity. Pharmaceutical industries could be encouraged to provide additional means.

“*The government must obtain alternative funding instead of depending on the state budget to implement HTA*. *I think the pharmaceutical industry can actually be asked to provide additional income in order to finance experts or researchers of HTA studies”*. [Policymaker 2]

The pharmaceutical industry representative suggested the creation of a clear framework for performing HTA implementation. The healthcare providers recommended that the government would provide more training and seminars to improve the capability of human resources regarding HTA. The healthcare professionals similarly suggested opening more HTA departments in universities in Indonesia. Finally, patients expected the government and all stakeholders to have a good collaboration in terms of introducing the implementation of HTA in Indonesia.

“*The government through the health ministry should establish guidelines to have a clear path to implement HTA”* [Pharmaceutical Industry]

*“The government should encourage universities to open HTA departments*. *So*, *we can learn the topics of HTA in a good curriculum”*. [Pharmacist 1]

*I hope all stakeholders can work together and find a good solution to start the implementation of HTA in Indonesia”*. [Patient 6]

#### Theme 6: Promoting factors for the implementation of HTA

The participants identified several promoting factors for the implementation of HTA. First of all, the main factor mentioned by all stakeholder’s category was that the JKN-KIS aims to achieve UHC. Several stakeholders perceived that the use of HTA is suitable for countries that are on their way to implement UHC. A second promoting factor mentioned was that the use of HTA is already in the regulation for implementing JKN-KIS, in particular to select medicines which will becovered by the BPJS-Kesehatan. Therefore, the use of HTA is mandatory in developing the list of medicines in the e-Catalogue and the NF. A third promoting factor was the use of the current e-Catalogue and the NF without HTA implementation have not helped sufficiently to reduce out-of-pocket payments for medicines.

"*The supporting factor is that we are implementing an international scale program*, *which is Universal Health Coverage*. *The HTA program is highly recommended World Health Organization for countries implementing UHC program*.*”* [Physician 6]

“*The regulation of Indonesia stated that HTA should be conducted for selecting the healthcare services needed*. *Indonesia is lucky since it has a legal aspect*, *whereas Vietnam does not have this*. *In some European countries it is also not present”*. [Policymaker 2]

“*We need a list of medications which were well selected at the NF*. *This was to avoid the doctors prescribed medicines not listed in the NF and also to prevent patients from spending money because they have to buy medicines that are not covered by BPJS Kesehatan”*. [Pharmacist 5]

## Discussion

This study provides insight into the current implementation of HTA in Indonesia in the development of the e-Catalogue and the National Formulary as medicines policies, as perceived by multiple stakeholders. To the best of our knowledge, no previous interview-based studies on this topic were performed in South East Asia. All interviewees showed a positive attitude towards the application of HTA to the e-Catalogue and the NF. The interviewees expected HTA could optimize the use of the e-Catalogue and the NF to reduce out-of-pocket payments and, subsequently, to achieve UHC in Indonesia. Furthermore, the stakeholders identified the advantages of applying HTA to both the e-Catalogue and the NF were greater than the disadvantages. However, interviewees mentioned that the application of HTA has the potential to increase costs for the development of the e-Catalogue and the NF. Specifically, this study clarifies the barriers, possible solutions, and facilitators of HTA implementation.

Interestingly, some interviewees perceived that the use of HTA would increase the cost for developing the e-Catalogue and the NF. However, other participants mentioned that the use of HTA for the e-Catalogue and the NF could improve efficiency. Our previous study [18] has revealed that the e-Catalogue and the NF have not been fully utilized in the healthcare facilities and were often ignored by stakeholders. This implies that unnecessary spendings have been allocated for producing these two medicine policies. For the e-Catalogue, HTA might support the setting of minimum prices as one of the problems mentioned was that the final tendered prices of medicines were too low and might jeopardize quality and distribution of medicines. Such prices would then reflect reasonable price levels for each medicine, in terms of costs per quality adjusted life years gained [24]. In addition, interviewees mentioned that the use of HTA in the NF might help to provide transparency and evidence for selecting medicines listed in the NF, and that this could improve its acceptance and use by stakeholders. Several LMICs aiming to achieve UHC have to consider a way to limit or choose the available healthcare services and HTA is one of the tools to support reimbursement package decision making. Many exemplary countries, such as Thailand, China, and Australia show how the use of HTA is helpful in selecting the medicines listed in their medicine reimbursement list. Thus, a more effective way of achieving UHC is achieved [6].

Several barriers to implement HTA were identified and reported in the results. The two most important barriers mentioned were a lack of local human resources and a lack of financial incentives. Previous studies [25] based on surveys stated that these barriers were the major barriers in 19 LMICs, including Vietnam as Indonesia's neighboring country. In addition, for other neighboring countries, such as Philipines and Malaysia, capacity building and financial incentives were also identified as significant barriers for implementing HTA in their medicine policies [26–28]. In the current study, the interviewees offered several possible solutions to overcome these obstacles. An interesting solution suggested by the NHI agency was that the HTA committee could establish a network with other countries that have a well-established system, and with international HTA organizations. In response to financial barriers, the authors support a solution suggested by the HTA committee that the Government of Indonesia could provide an alternative funding instead of depending on the state budget. The interviewees recognized the need of HTA but also wondered how it should be funded. We think the implementation as achieved by the HTA committee of Thailand may serve as an example. The Committee obtains funding from the Thai Health Promotion Foundation, an institution established by the Ministry of Health of Thailand to collect health funding through two percents surcharge levied on the excise tax of alcohol and tobacco. This institution also obtains its funding from pharmaceutical companies and international HTA agencies [29].

The main important factor mentioned to support HTA implementation in the e-Catalogue and the NF was a mandate in the Presidential Decree [30], which stated that the development of medicine policies must be based on the HTA study. This implies the HTA must be applied in the e-Catalogue and the NF development. Based on a previous international study [31], this implication can lead to a continuous development of the capacity building for local human resources.

Conducting interviews allowed the researcher to obtain detailed information about personal feelings, perceptions and opinions from the participants indepedently from other group members. Moreover, all ambiguities and incomplete answers could be clarified and followed up. Several steps were taken to ensure a good research practice during the data compilation and analysis stages. The sampling processs used to select participants and the interview guide were compared with recommendations published in the COREQ [22].

Though a variety of stakeholders were interviewed in this study, not all groups of stakeholders were equally large and it turned out hard to find industry representatives willing to participate. Notably, only one pharmaceutical industry representative agreed to participate. Nonetheless, the interviewee was relatively experienced and was a leader in several pharmaceutical associations in Indonesia.

While saturation was reached at the level of the total group, it could not be ensured for smaller groups of stakeholders, namely industry and policy makers. Also, this research contained interviews with a specific group of patients, namely from the Prolanis. The main reason for this was that participants from this group were relatively easy to find and recruit. These patients affiliated to the Prolanis group were considered as being able to provide more information than other patients since they had already routinely consumed medicines and had actively used public health insurance in Indonesia for a significant time period. It is relevant to note that quite a few people did not feel comfortable to participate or withdrew their consent after having seen the questions for the interview. This may indicate that a substantial part of stakeholders is not very familiar with the concepts discussed during the interviews and/or considers the topics as hard to discuss.

Recognizing the current barriers and facilitators identified to apply HTA in the development of the e-Catalogue and the national formulary could assist decision makers in developing a blueprint for the further implementation of HTA in Indonesia. This could also be relevant for international policymakers in other LMICs with similar characteristics and ambition to achieve UHC.

## Conclusions

Several barriers are currently hampering the implementation of HTA to support medicine pricing and reimbursement policies in Indonesia. However, solutions for these issues are under consideration and facilitators do exist. The major barriers to the implementation of HTA are a lack of capacity of local human resources and a lack of (financial) incentives. Possible solutions to address these issues would be to establish a network with other countries that have a well-established system, and with international HTA organizations. A major opportunity to support the implementation of HTA in the e-Catalogue and the NF is the existing legal framework to implement HTA in medicine policies of the JKN-KIS program in Indonesia.

## Supporting information

S1 Fig Conceptual framework. (PDF)

S1 Table Initial list of themes and of questions. (PDF)

S2 Table Overview of the recruitment process. (PDF)

S3 Table Saturation checklist on sub-themes. (PDF)

S4 Table Raw materials. (XLSX)

S1 File Interview protocol. (PDF)

S2 File Informed consent. (PDF)

S3 File A formal waiver statements. (PDF)
