# Supplementary material for: The implementation of HTA in medicine pricing and reimbursement policies in Indonesia: Insights from multiple stakeholders
Source: PLoS One. 2019 Nov 27;14(11):e0225626. doi: 10.1371/journal.pone.0225626 (PMC6881021; doi:10.1371/journal.pone.0225626)
Supplement: S1 Table — (PDF) [file pone.0225626.s002.pdf]

S1 Table. Initial list of themes and of questions

No	Themes	No	Questions
**1**	The development of medicine policy and national health insurance	1	Do you know how long HTA Committee, NF Committee, and NHI Agency existed?
		2	How many committee members?
		3	What are their professions?
		4	What are their duties?
		5	Why are they existed?
		6	What is the fuction of them?
		7	What is the role of them to support UHC?
		8	How the NHI transform from the previous Health Insurance (Askes, Jamkesmas, etc)?
		9	How many people are covered by NHI Agency currently?
**2**	Medicine Policy for UHC	10	What should medicine policy(s) be created to support UHC?
		11	Are you involved in creating NF and other medicine policy to support UHC?
**3**	Users Perspective	12	Do you know how the National Formulary is created?
		13	Do you agree with the NF?
		14	Do you have policy recommendations to maximize the use of HTA?
		15	Do you have other policy recommendations for prescribing drugs?
**4**	Obstruction and promoting factors of HTA	16	What is/are the advantages using HTA in Medicine Policy?
		17	What is/are the disadvantages using HTA in Medicine Policy?
		18	What is/are the facilitators implementing HTA in medicines?
		19	What is/are the barriers implementing HTA in medicines?
		20	What should other stakeholders do to solve them? And Which?
		21	What should the government do?
**5**	The biggest burden of disease and medicine expenditure	22	What is the largest disease in Indonesia
		23	How much money is allocated for medicines in National Health System?
		24	What medicine are allocated more? (Why)
**6**	Prescription outside of NF	25	Have you ever received drugs prescription outside NF (How frequently and why)?
		26	which medicines do you receive often inside NF?
		27	Do you agree when doctors prescribe medicines outside NF? Why?
