# Supplementary material for: The implementation of HTA in medicine pricing and reimbursement policies in Indonesia: Insights from multiple stakeholders
Source: PLoS One. 2019 Nov 27;14(11):e0225626. doi: 10.1371/journal.pone.0225626 (PMC6881021; doi:10.1371/journal.pone.0225626)
Supplement: S2 Table — (PDF) [file pone.0225626.s003.pdf]

S2 Table. Overview of the recruitment process

Type of stakeholders	Sent letter	Reaction to letter	Interviews	Cancelled participation before interview, after receiving interview guide	Cancelled participation during interview	Not possible to schedule interview time	No reaction within 10 months
WHO members	2	2	1	0	0	1	0
HTA committee	3	2	2	0	0	0	1
National formulary committee	3	1	1	0	0	0	2
NHI Agency	3	3	2	0	0	1	0
Pharmaceutical industry	4	3	1	0	0	2	1
Physicians	12	10	6	1	3	0	2
Pharmacists	15	15	6	5	4	0	0
Patients	9	9	6	3	0	0	0
**TOTAL**	**51**	**45**	**25**	**9**	**7**	**4**	**6**
