# Supplementary material for: The implementation of HTA in medicine pricing and reimbursement policies in Indonesia: Insights from multiple stakeholders
Source: PLoS One. 2019 Nov 27;14(11):e0225626. doi: 10.1371/journal.pone.0225626 (PMC6881021; doi:10.1371/journal.pone.0225626)
Supplement: S3 Table — (PDF) [file pone.0225626.s004.pdf]

S3 Table. Saturation checklist on sub-theme

No	Themes	Policy Makers						Medicine Supplier	Physicians						Pharmacist						Patients					
		1	2	3	4	5	6	1	1	2	3	4	5	6	1	2	3	4	5	6	1	2	3	4	5	6
1	**Attitude**																									
	Agree	+	+	+	+	+	+	+	+	+	+	+	+	+	+	+	+	+	+	+	+	+	+	+	+	+
2	**Advantages**																									
	To Provide scientific evidence	+	+	+	+	+	+	+	+	+		+	+	+			+	+								
	To Provide a ground for fair pricing in the e-Catalogue							+		+				+		+			+					+		
	To reassure the medicines listed in the NF were the best choice	+	+	+	+		+		+	+	+	+	+	+	+	+	+		+	+	+	+	+		+	+
3	**Disadvantages**																									
	Increase the cost of developing the e-Catalogue and the NF	+									+	+		+			+	+	+				+			
	Complicated bureaucracy	+		+			+																			
	Lengthy Process for renewing medicines listed in the e-Catalogue and the NF	+		+			+		+	+	+			+			+									
	No disadvantage		+		+	+		+							+	+				+	+	+			+	+
4	**Barriers**																									
	A lack of capability of local human resources	+	+	+	+	+	+	+	+		+	+	+	+	+			+	+	+						
	A lack of financial incentives	+	+	+	+	+	+					+					+				+	+	+	+	+	+
	A lack of a clear framework	+	+				+	+		+				+	+	+		+								
	Insufficient data	+	+				+								+			+	+							
5	**Possible solutions**																									
	To establish (inter)national network to build up capacity	+	+	+	+		+			+		+		+							+	+	+	+	+	+
	To introduce a clear HTA framework	+	+			+	+	+	+		+		+	+		+	+	+	+	+	+					
	To open HTA department, training, and association	+	+	+	+					+	+	+	+	+	+					+						
6	**Facilitating factors**																									
	The ambition to achieve UHC	+	+	+		+	+		+	+	+	+	+	+			+		+	+	+		+			
	The presence of legal framework to implement HTA in the e-Catalogue and the NF	+	+	+	+	+	+					+			+	+		+	+	+					+	+
	The demand for appropriate medicine policies.	+	+					+														+		+		
