# Supplementary material for: The implementation of HTA in medicine pricing and reimbursement policies in Indonesia: Insights from multiple stakeholders
Source: PLoS One. 2019 Nov 27;14(11):e0225626. doi: 10.1371/journal.pone.0225626 (PMC6881021; doi:10.1371/journal.pone.0225626)
Supplement: S2 File — (PDF) [file pone.0225626.s007.pdf]

S2 File. Informed consent

Lembar penjelasan partisipan

Peluang dan tantangan kebijakan obat pada program JKN-KIS untuk mencapai Cakupan Perlindungan Semesta / *Universal Health Coverage* (UHC)

Saya Riswandy Wasir, Mahasiswa Program Pascasarjana (S3) Departemen Epidemiologi, Univerity Medical Center Groningen (UMCG), University of Groningen, Netherlands. Saat ini saya sedang melakukan penelitian yang berjudul "**peluang dan tantangan kebijakan obat pada program JKN-KIS untuk mencapai cakupan perlindungan semesta**.

Pemerintah Indonesia telah menerapkan skema asuransi kesehatan nasional (JKN-KIS) yang terintegrasi dimulai pada tahun 2014. Asuransi kesehatan yang dikelolah oleh BPJS-Kesehatan dimaksudkan sebagai pengganti seluruh asuransi kesehatan publik sebelumnya. Peta jaminan kesehatan nasional yang dirilis oleh pemerintah menyatakan bahwa program JKN-KIS diupayakan mencapai cakupan perlindungan semseta/ *Universal Health Coverage* (UHC) pada tahun 2019. Organisasi Kesehatan Dunia/ *World Health Organization* (WHO) menyatakan bahwa bukan hanya kepesertaan yang menjadi indikator tercapainya cakupan perlindungan semesta, tetapi juga kualitas pelayanan.

Berbagai upaya telah dilakukan oleh pemerintah dan para pemangku kebijakan untuk meningkatkan mutu pelayanan JKN-KIS. Pada sektor obat, pemerintah Indonesia telah menerapkan formularium nasional. Manfaat Fornas yaitu sebagai acuan penetapan penggunaan obat dalam JKN-KIS, serta meningkatkan penggunaan obat yang rasional, dapat juga mengendalikan mutu dan biaya pengobatan, serta mengoptimalkan pelayanan kepada pasien Namun, Hasil evaluasi awal yang dilakukan oleh Direktorat Jenderal Bina Farmasi dan Alat Kesehatan (Dirjen Binfar dan Alkes) memperlihatkan bahwa kesesuaian obat yang digunakan di rumah sakit dengan formularium nasional berkisar antara 60 – 86 %. Hal ini memperlihatkan bahwa masih ditemukan *cost-sharing*, suatu kondisi dimana peserta JKN-KIS harus mengeluarkan biaya untuk pembayaran pelayanan kesehatan termasuk obat. Salah satu Indikator tercapainya cakupan perlindungan semesta (UHC) menurut WHO adalah tidak adanya *cost-sharing*.

Kami, melakukan penelitian ini untuk mengidentifikasi peluang dan tantangan kebijakan obat pada program JKN-KIS untuk mencapai cakupan perlindungan semesta. Penelitian ini akan melibatkan beberapa pemangku kepentingan yang dianggap memiliki peran dalam membuat kebijakan, mengimplementasikan serta merasakan dampak dari kebijakan tersebut antara lain sebagai berikut:

No	Partisipan	Peran
1	Organisasi Kesehatan Dunia (WHO)	Pembuat Kebijakan skala Internasional
2	Komite HTA	Pembuat kebijakan skala nasional
3	Komite Formularium Nasional	Pembuat kebijakan skala nasional
4	BPJS-Kesehatan	Penyelenggara JKN-KIS dan Pembayar
5	Perusahaan Farmasi	Pemasok obat
6	Dokter	Penyedia pelayanan kesehatan
7	Apoteker	Penyedia pelayanan kesehatan
8	Pasien	Peserta JKN-KIS/ Pengguna

Penelitian akan dilaksanakan dengan metode wawancara bebas terpimpin (*semi-structured interview)*. Daftar pertanyaan, surat kesediaan berpartisipasi (*informed consent*), dan pedoman wawancara akan diberikan oleh peneliti kepada partisipan sehari sebelum wawancara atau pada waktu yang telah ditentukan bersama. Durasi waktu pada saat melaksanakan wawancara mendalam yaitu sekitar 1 (satu) jam.

A. Kesukarelaan untuk ikut penelitian

Bapak/ Ibu/ Saudara bebas memilih keikutsertaan dalam penelitian ini tanpa ada paksaan. Bila bapak/ ibu/ saudara sudah memutuskan untuk ikut dapat juga mengundurkan diri setiap saat tanpa dikenai denda ataupun sanksi apapun.

B. Prosedur Penelitian

Apabila Bapak/ Ibu/ Saudara bersedia berpartisipasi dalam penelitian ini, diminta menandatangani lembar persetujuan ini rangkap dua, satu untuk Bapak/ Ibu/ Saudara simpan, dan satu untuk peneliti. Prosedur selanjutnya adalah :

1. Penelitian akan dilaksanakan dengan metode wawancara terpimpin (*semi-structured Interview*) sesuai dengan kesepakatan dan kenyamanan bapak/ ibu/ saudara menggunakan alat perekam.
2. Peneliti akan mentranskrip hasil wawancara setelah melaksanakan interview dengan partisipan.
3. Hasil transkrip akan diperlihatkan kembali kepada partisipan untuk mengklarifikasi hasil wawancara.
4. Hasil transkrip yang telah disepakati antara peneliti dan partisipan akan diproses oleh peneliti beserta tim peneliti lainnya untuk kemudian dijadikan data kualitatif yang akan di publikasi.

C. Kewajiban partisipan

Sebagai partisipan, Bapak/Ibu/Saudara berkewajiban mengikuti aturan atau petunjuk penelitian seperti yang tertulis di atas. Bila ada yang belum jelas, Bapak/ Ibu/ Saudara bisa bertanya lebih lanjut kepada peneliti.

D. Risiko

Penelitian ini tidak menyebabkan risiko yang berbahaya, karena peneliti tidak melakukan intervensi apapun. Risiko ketidaknyamanan atau dampak yang mungkin akan diterima yaitu merasa bosan dengan banyaknya pertanyaan- pertanyaan dari peneliti dan kerugian waktu selama diwawancarai oleh peneliti.

E. Manfaat dan Keuntungan

Manfaat dan keuntungan yang bapak/ ibu/ saudara dapatkan adalah kesempatan dan kebebasan untuk menjelaskan mengenai apa yang anda rasakan terhadap kebijakan obat pada program JKN-KIS. Penelitian ini nantinya akan diupayakan menjadi masukan terhadap pengambil kebijakan dalam meningkatkan sektor kesehatan nasional.

F. Kerahasiaan

Semua informasi yang berkaitan dengan identitas partisipan akan dirahasiakan dan hanya akan diketahui oleh peneliti. Data hasil penelitian akan disimpan dan didokumentasikan oleh peneliti serta hanya digunakan untuk kepentingan ilmiah. Hasil penelitian akan dipublikasikan tanpa identitas partisipan.

G. Kompensasi

Bapak/ ibu/ saudara akan mendapatkan souvenir sebagai wujud terima kasih peneliti atas partisipasinya dalam penelitian ini.

H. Informasi Tambahan

Bapak/ ibu/ saudara diberi kesempatan untuk menanyakan semua hal yang belum jelas sehubungan dengan penelitian ini. Bila sewaktu-waktu terjadi kekeliruan atau membutuhkan penjelasan lebih lanjut, Bapak/ ibu/ saudara dapat menghubungi Riswandy Wasir pada no HP: +62-81225835007 atau bisa juga dengan mengirimkan email: riswandy.wasir@yahoo.com

Lembar persetujuan partisipan

Saya yang bertandatangan dibawah ini:

Nama : ....

Alamat : ....

Nomor Hp : ....

E-mail : ....

Umur : ....

Pekerjaan : ....

Pendidikan Terakhir : ....

Peran partisipan :

1. Anggota Organisasi Kesehatan Dunia / *World Health Organization* (WHO)
2. Komite Penilaian Teknologi Kesehatan / Health Technology Assessment (HTA)
3. Komite Formularium Nasional
4. Instansi Penyelenggara Asuransi Kesehatan Nasional
5. Perusahaan Farmasi
6. Dokter
7. Apoteker
8. Pasien

Menyatakan bahwa, semua penjelasan tersebut telah disampaikan kepada saya sebagai partisipan dan semua pertanyaan saya telah dijawab oleh peneliti. Saya mengerti bahwa bila memerlukan penjelasan, saya dapat menanyakan kepada saudara Riswandy Wasir sebagai peneliti utama.

Dengan menandatangani formulir ini, saya setuju untuk ikut serta dalam penelitian ini

Tanggal:

Tanda Tangan Partisipan

Nama Jelas: …………………………….

Tanggal:

Tanda Tangan Peneliti

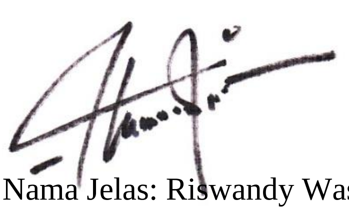

Nama Jelas: Riswandy Wasir
