# Supplementary material for: The implementation of HTA in medicine pricing and reimbursement policies in Indonesia: Insights from multiple stakeholders
Source: PLoS One. 2019 Nov 27;14(11):e0225626. doi: 10.1371/journal.pone.0225626 (PMC6881021; doi:10.1371/journal.pone.0225626)
Supplement: S3 File — (PDF) [file pone.0225626.s008.pdf]

S3 File. A formal waiver statements

P.O. Box 30 001, 9700 RB Groningen, The Netherlands

University Medical Center Groningen

Medical Ethics Review Board

Phone +31(0)50 361 42 04
Fax +31 (0)50 361 43 51
E-mail metc@umcg.nl
Website: metcgroningen.nl

Ref. M18.236401

To
R. Wasir, MSc.
University Medical Center Groningen
Department of Epidemiology FA 40

Date: 30th August 2018
Subject: **The role of Health Technology Assessment for Pharmaceutical Pricing and Reimbursement Policy in Indonesia.**

Dear Sir,

I, the undersigned, declare that the submission entitled *"The role of Health Technology Assessment for Pharmaceutical Pricing and Reimbursement Policy in Indonesia"* by Wasir e.a., fulfils all the requirements for patient anonymity and is in agreement with regulations of our University Hospital for publication of patient data.

Sincerely,

W.A. Kamps MD PhD
chairman

cc *-T.L. Feenstra MD, University Medical Center Groningen, Department of Epidemiology FA 40*
-E. Buskens, MD Ph.D, University Medical Center Groningen, Department Epidemiology FA40

umcg
